# Performance of Coarse Graining in Estimating Polymer Properties: Comparison with the Atomistic Model

**DOI:** 10.3390/polym12020382

**Published:** 2020-02-08

**Authors:** Ryota Miwatani, Kazuaki Z. Takahashi, Noriyoshi Arai

**Affiliations:** 1 Department of Mechanical Engineering, Kindai University, 3-4-1 Kowakae, Higashiosaka, Osaka 577-8522, Japan; miwatani.r.d5@gmail.com; 2 Research Center for Computational Design of Advanced Functional Materials, National Institute of Advanced Industrial Science and Technology (AIST), Central 2, 1-1-1 Umezono, Tsukuba, Ibaraki 305-8568, Japan; 3 Department of Mechanical Engineering, Keio University, 3-14-1 Hiyoshi, Yokohama, Kanagawa 223-8522, Japan; arai@mech.keio.ac.jp

**Keywords:** polymer physics, molecular dynamics, coarse-grained model, atomistic model

## Abstract

Combining atomistic and coarse-grained (CG) models is a promising approach for quantitative prediction of polymer properties. However, the gaps between the length and time scales of atomistic and CG models still need to be bridged. Here, the scale gaps of the atomistic model of polyethylene melts, the bead–spring Kremer–Grest model, and dissipative particle dynamics with the slip-spring model were investigated. A single set of spatial and temporal scaling factors was determined between the atomistic model and each CG model. The results of the CG models were rescaled using the set of scaling factors and compared with those of the atomistic model. For each polymer property, a threshold value indicating the onset of static or dynamic universality of polymers was obtained. The scaling factors also revealed the computational efficiency of each CG model with respect to the atomistic model. The performance of the CG models of polymers was systematically evaluated in terms of both the accuracy and computational efficiency.

## 1. Introduction

Because of scientific and industrial interest, entangled polymer materials have been studied for many years. However, accurate quantitative prediction of polymer properties is still a long-standing issue because of the slow relaxation nature of the polymer dynamics far from the nanoscale of the molecular architecture [1,2,3]. Atomistic molecular dynamics (MD) simulations trace the dynamics of polymer molecules using realistic length and time units in a straight forward manner, but the simulation time (up to a few microseconds) is not sufficient to cover polymer relaxation (up to seconds, minutes, hours, months, or years), even using current high-performance computers. Thus, coarse graining of molecular models and simulations was developed to cover larger timescales [4,5,6,7,8,9]. To accelerate simulations, in most cases, the number of degrees of freedom is reduced and a Langevin equation of motion is often used. Importantly, the realistic units of length, time, temperature, and so forth are lost during such coarse graining, and thus coarse-grained (CG) MD (CGMD) simulations only return qualitative values. These “scale gaps” make it difficult to evaluate the accuracy of the estimated polymer properties and determine how much MD simulations are accelerated using CG models. To solve this problem and quantitatively evaluate CG models, the results of CGMD simulations should be rescaled by using realistic units. A general way to rescale the results is application of the scaling law of polymer dynamics experimentally determined for polymer materials [1,2,10,11,12,13,14,15,16,17]. For simple straight-chain polymers, the scaling law can be determined regardless of the difference of the chemistry of the molecular architecture. Importantly, the above character of the law can be applied to different molecular models of polymers even though each model has a different concept, such as the presence/absence of chemistry, employment of the Newton/Langevin equation of motion, and so forth. This was investigated in several previous reports. Hamandaris et al. [18] compared the polymer dynamics of the atomistic polystyrene (PS) model with that of the CG PS model. The CG PS model was rescaled by a temporal scaling factor determined by comparison of the unentangled short chain dynamics. Masubuchi et al. [19] investigated the compatibility of the dynamical and static polymer properties among different models of the multi-chain slip-link model, multi-chain slip-spring model, and bead–spring model. Polymer diffusion of the three models coincided by using spatial and temporal scaling factors, showing the compatibility of the models. Takahashi et al. [20] compared the polymer dynamics of the atomistic PE model with that of the bead-spring model. They determined a set of spatial and temporal scaling factors to successfully rescale the results of the bead–spring model, but the scaling behavior around the onset of entanglement was different for each polymer property. This quantifies the applicable range of bridging between the models and suggests the possibility of further improvements for CG models. Nevertheless, few studies evaluated both the accuracy and computational efficiency of CG models rescaled by the above scheme.

In this work, we investigated the scale gaps of the atomistic model of polyethylene (PE) melts, bead–spring Kremer–Grest (KG) model [5,8], and dissipative particle dynamics (DPD) with slip-spring model [21]. A single set of spatial and temporal scaling factors was determined between the atomistic model and each CG model. The results of the CGMD simulations were rescaled using the set of scaling factors and compared with those of the atomistic model. Threshold values that indicate the onset of static and dynamic universality among the polymer models were obtained for almost all of the results of comparison of the polymer properties, showing the accuracy of the CG models. The computational efficiency of each CG model was quantitatively estimated with respect to the atomistic model. The performance of the CG models of polymers was systematically and quantitatively evaluated in terms of both the accuracy and computational efficiency.

## 2. Methodology

### 2.1. Bead–Spring KG Model

In this work, the bead–spring model for polymer chains developed by Kremer and Grest [8] was used as the coarse-grained model without chemical parameters. The model consists of simple beads whose non-bonded interactions are described by the following repulsive LJ potential:

(1) $$\begin{array}{ccc} u_{\mathrm{LJ}}\left(r\right) & = & \left\{\begin{array}{c} 4\varepsilon \left[{\left(\frac{\sigma }{r}\right)}^{12}-{\left(\frac{\sigma }{r}\right)}^{6}+\frac{1}{4}\right]\;\left(r/\sigma <2^{1/6}\right)\\ 0\;(r/\sigma >2^{1/6}) \end{array}\right., \end{array}$$

where ε=1 is the unit of energy, σ=1 is the unit of length, and *r* is the distance between two beads. The bonds between beads are described by the following finite extensible nonlinear elastic (FENE) potential:

(2) $$\begin{array}{ccc} u_{\mathrm{FENE}}\left(r\right) & = & \left\{\begin{array}{c} -\frac{kR_{0}^{2}}{2}\ln{\left(1-\frac{r}{R_{0}}\right)}^{2}\;\left(r<R_{0}\right)\\ \infty \;(r>R_{0}) \end{array}\right., \end{array}$$

where $k=30\;\varepsilon /\sigma^{2}$ is the spring constant and $R_{0}=1.5\;\sigma$ is the maximum extension of the spring. For polymer motion in the KG model, the following Langevin-type equation is used:

(3) $$\begin{array}{ccc} m_{\mathrm{KG}}{\ddot{r}}_{i} & = & -\nabla \sum_{i\neq j}\left[u_{\mathrm{LJ}}\left(r_{ij}\right)+u_{\mathrm{FENE}}\left(r_{ij}\right)\right]-\Gamma {\dot{r}}_{i}+W\left(t\right), \end{array}$$

(4) $$\begin{array}{ccc} \langle W\left(t\right)\cdot W\left(t^{\prime }\right)\rangle & = & 6k_{B}T\delta_{ij}\delta \left(t-t^{\prime }\right), \end{array}$$

where $m_{\mathrm{KG}}$ is the mass of the KG bead, ***r*** are the coordinates of the bead, Γ is an arbitrary friction constant, W(t) is a random force, $k_{B}$ is Boltzmann’s constant, and $T=\varepsilon /k_{B}$ is the temperature. $m_{\mathrm{KG}}$, $k_{B}$, and *T* were set to 1, and Γ was set to 0.5. All of the simulations using the KG model were performed using the NVT ensemble. The monomer density was $0.85\;m_{\mathrm{KG}}/\sigma^{3}$. The velocity Verlet integrator [22] was used with three-dimensional periodic boundary conditions and a time step of $\Delta t_{\mathrm{KG}}=0.006\;\tau_{\mathrm{KG}}$, where $\tau_{\mathrm{KG}}$ is the time unit of the KG model. The number of KG beads ($N_{\mathrm{KG}}$) varied from 20 to 250. For $N_{\mathrm{KG}}=20$–80, equilibrium simulations with a total of $2\times 10^{8}$ time steps were performed for three independent initial structures. For $N_{\mathrm{KG}}=100$–250, equilibrium simulations with a total of $6\times 10^{8}$ time steps were performed for six independent initial structures. The total number of beads in the system ($N_{\mathrm{total}}$) was fixed at about 25,000. Each KG MD (KGMD) simulation system was equilibrated prior to data acquisition.

### 2.2. DPD with the Slip-Spring Model

We used the DPD method [6,23,24] to reproduce the behavior of the polymer melt. The DPD method was proven to be an effective mesoscopic simulation tool to study fluid events occurring at millisecond timescales and micrometer length scales via by tracking the motion of coarse-grained particles (composed of a group of atoms or molecules). Many researchers studied the thermodynamic and morphological behavior of polymers using the DPD method, for example, self-assembly of dendritic copolymers [25,26,27], micelle fusion or vesicle formation [28,29], and nanocomposite systems [30,31,32,33].

The fundamental equation in the DPD method is Newton’s equation of motion. Newton’s equation of motion for particle *i* is given by

(5) $$m_{i}\frac{dv_{i}}{dt}=f_{i}=\sum_{j\neq i}F_{ij}^{C}+\sum_{j\neq i}F_{ij}^{D}+\sum_{j\neq i}F_{ij}^{R}\;\;,$$

where v is the velocity vector, $F^{C}$ is the conservative force, $F^{R}$ is the pairwise random force, and $F^{D}$ is the dissipative force. The conservative force is softly repulsive:

(6) $$F_{ij}^{C}=\left\{\begin{array}{cc} -a_{ij}\left(1-\frac{\left|r_{ij}\right|}{r_{c}}\right)n_{ij}, & \left|r_{ij}\right|\leq r_{c}\\ \;\;\;\;\;\;\;\;\;\;\;\;\;\;\;0, & \left|r_{ij}\right|>r_{c}\;\;, \end{array}\right.$$

where $r_{ij}=r_{j}-r_{i}$ and $n_{ij}=r_{ij}/\left|r_{ij}\right|$. Here, $a_{ij}$ is a parameter to determine the magnitude of the repulsive force between particles *i* and *j*, and $r_{c}$ is the cutoff distance. The random force $F_{ij}^{R}$ and dissipative force $F_{ij}^{D}$ are given by

(7) $$F_{ij}^{R}=\left\{\begin{array}{cc} \zeta \omega^{R}\left(\left|r_{ij}\right|\right)\zeta_{ij}\Delta t^{-1/2}n_{ij}, & \left|r_{ij}\right|\leq r_{c}\\ \;\;\;\;\;\;\;\;\;\;\;\;\;\;\;0, & \left|r_{ij}\right|>r_{c} \end{array}\right.$$

and

(8) $$F_{ij}^{D}=\left\{\begin{array}{cc} -\gamma \omega^{D}\left(\left|r_{ij}\right|\right)\left(n_{ij}\cdot v_{ij}\right)n_{ij}, & \left|r_{ij}\right|\leq r_{c}\\ \;\;\;\;\;\;\;\;\;\;\;\;\;\;\;0, & \left|r_{ij}\right|>r_{c} \end{array}\right.$$

respectively, where $v_{ij}=v_{j}-v_{i}$, ζ is the noise parameter, γ is the friction parameter, and $\zeta_{ij}$ is a random number based on the Gaussian distribution. Here, $\omega^{R}$ and $\omega^{D}$ are *r*-dependent weight functions:

(9) $$\omega^{D}\left(r\right)={\left[\omega^{R}\left(r\right)\right]}^{2}=\left\{\begin{array}{cc} {\left[1-\frac{\left|r_{ij}\right|}{r_{c}}\right]}^{2}, & \left|r_{ij}\right|\leq r_{c}\\ \;\;\;\;0, & \left|r_{ij}\right|>r_{c}\;\;. \end{array}\right.$$

The temperature is controlled by a combination of dissipative and random forces. The noise parameter ζ and friction parameter γ are connected by the fluctuation–dissipation theorem:

(10) $$\zeta^{2}=2\gamma k_{B}T.$$

Usually, reduced units are used for reporting DPD results. The DPD unit of length is the cutoff radius ($r_{c}$), the unit of mass is the DPD bead mass ($m_{\mathrm{DPD}}$), and the unit of energy is $k_{B}T$. Hence, $r_{c}$ = $m_{\mathrm{DPD}}$ = $k_{B}T$ = 1.

In this work, the number of DPD beads $N_{\mathrm{DPD}}$ was varied from 5 to 100. Each particle and its nearest neighbor in the polymer are connected by a harmonic spring described by

(11) $$F_{ij}^{S}=-k\left(\left|r_{ij}\right|\right)n_{ij}$$

with a spring constant of *k* = 2 $k_{B}T/r_{c}^{2}$ [21].

All of the simulations were performed with the NVT ensemble. The particle density and temperature were set to 3.0 $r_{c}^{-3}$ and 1.0 $k_{B}T$, respectively. Periodic boundary conditions were applied in all three dimensions. In the DPD simulation, the thermostat is achieved by pairwise random and dissipative forces. These forces are coupled by the fluctuation-dissipation theorem [24]. The noise amplitude ζ and friction coefficient γ are included in the dissipative and random forces, respectively. They satisfy ζ = $\sqrt{2\gamma k_{B}T}$, which reproduces the canonical ensemble. ζ and γ were set to 3.0 and 4.5, respectively.

The total number of beads in the system ($N_{\mathrm{total}}$) is 3000 to 24,000 depending on the chain length. In our simulations, the interaction parameters in Equation (1) are described by $a_{ij}$ = 25 $k_{B}T/r_{c}$.

In the DPD method, a soft potential is also used as the interaction between particles. However, it is known to be unable to reproduce entanglement effects [34]. To capture the entanglement behavior of the polymer, slip-springs [21,35,36] were introduced with the DPD polymer chains. The details of the procedure are given elsewhere [21,36]. The number of slip-springs is 300 to 2400, namely a slip-spring is placed for every ten beads.

Each run was performed for $10^{7}$ time steps with a time step $\Delta t_{\mathrm{DPD}}=0.06\;\tau_{\mathrm{DPD}}$, where $\tau_{\mathrm{DPD}}$ is the time unit for DPD with the slip-spring model, to reproduce the equilibrium polymer melt. The reported data are the average over the last $5\times 10^{6}$ time steps.

### 2.3. Atomistic Model of PE

Most accurate classical MD simulations are performed using all-atom (AA) molecular models. However, it is often beyond the capability of current computers to trace the slow polymer motion of the AA model up to a meaningful timescale [18]. Thereforem united-atom (UA) molecular models were developed and used as quantitative atomistic models. In UA models, a group of atoms, such as ${\mathrm{CH}}_{3}$, is merged into one UA particle whose non-bonded interactions are described by Lennard-Jones (LJ) potentials. Such UA models have three main benefits in terms of computational efficiency: (i) the degrees of freedom can be reduced, (ii) the time step for molecular motion can be extended, and (iii) computationally expensive Coulomb interactions can be omitted for UA particles that do not have an overall charge. Because of these benefits, UA models “accelerate” the timescale of AA molecular motion. A previous study showed that the dynamics of AA polystyrene (PS) systems is accelerated by about 120 times by using the UA PS model [18]. Importantly, this acceleration ratio between AA and UA models cannot be evaluated before direct comparison of the dynamics of the two simulations. This indicates that the UA model has a scale gap problem, like CG models. However, in terms of only computational efficiency, UA models are used for quantitative molecular simulations. In fact, UA models are widely used for MD simulations of polymers. For UA models of the common polymer PE, the anisotropic UA (AUA) [37], optimized potentials for liquid simulations (OPLS)-UA [38], and transferable potentials for phase equilibria force field (TraPPE)-UA [39] models are widely accepted [40,41,42,43,44,45,46]. MD simulations using UA PE models were recently performed for a wide range of polymer nanocomposites [47,48], polymer interfaces [49,50,51], ring polymers [52], nucleation of polymer droplets [53], the Fermi–Pasta–Ulam problem in realistic systems [54,55], and better understanding of the macroscopic mechanical properties [56,57,58,59,60].

In this work, the TraPPE-UA [39] model was used for the atomistic model of PE melts. The TraPPE-UA PE model consists of two different types of UAs (${\mathrm{CH}}_{3}$ and ${\mathrm{CH}}_{2}$), whose non-bonded interactions are described by LJ 12–6 potentials. The bond lengths between the UA particles are kept rigid using the LINCS algorithm [61]. The bond angle bending interactions are described using harmonic potentials. Rotations along the bonds in the aliphatic backbone are described using standard torsional potentials. These dihedral potentials are also used for the 1–4 non-bonded interactions. Atomistic MD (AMD) simulations using the above UA model were performed for PE melts with molecular weight (*M*) ranging from 422.8 to 3509g/mol. Please note that the critical molecular weight of the model was estimated to 983.9g/mol from the scaling law and primitive path analysis [20]. Therefore both unentangled and entangled motions of PE chains can be observed in this work. The MD package GROMACS [62] was used for the simulations. To obtain well-equilibrated initial atomistic structures, over 100ns long-time MD simulations with intermittent pressure increasing and temperature decreasing processes were performed with the constant particle number, pressure, and temperature (NPT) ensemble. The equilibrated systems were confirmed by comparison with the results of a previous study [46]. The product runs using the equilibrated initial structures were performed with the constant particle number, volume, and temperature (NVT) ensemble using the Nosé–Hoover thermostat [63,64]. The density and temperature were set to $0.650\;g/{\mathrm{cm}}^{3}$ and 500K, respectively. Non-bonded interactions were cut off beyond 1.2nm. The Verlet leapfrog integrator [65] was used with three-dimensional periodic boundary conditions and a time step of 2fs. For M=422.8–983.9g/mol, equilibrium simulations with a total of $5\times 10^{7}$ time steps (=100ns) were performed for three independent initial structures. For M=1405–2106g/mol, equilibrium simulations with a total of $2.5\times 10^{8}$ time steps (=500ns) were performed for six independent initial structures. For M=2807–3509g/mol, equilibrium simulations with a total of $5\times 10^{8}$ time steps (=1μs) were performed for six independent initial structures.

For easy understanding, here we state the two clear differences between AMD using TraPPE-UA and KGMD. The one is a force field. In TraPPE-UA, a bond, angle, torsion, and non-bonded repulsive and diffusive forces are quantitatively considered based on quantum and molecular mechanics. In contrast, KGMD considers only qualitative FENE-bond and non-bonded repulsive forces. The other is an equation of motion. In AMD, Newton’s equation of motion is simply but strictly used. In KGMD, Langevin equation is modified for polymer dynamics, and the arbitrary parameter Γ is employed for easy expression of friction between polymer chains.

### 2.4. Rescaling of the CG Models

Regarding the unentangled/entangled nature of polymer molecules, some scaling laws of the polymer properties have a critical molecular weight $M_{c}$ that indicates the change of the polymer dynamics. For simple explanation of the scaling laws, the Rouse model [66] and reptation model [1] were suggested for unentangled motion ($M<M_{c}$) and entangled motion ($M>M_{c}$), respectively. Thus, the scaling factor to rescale the CGMD simulation results can be determined by comparison of the unentangled or entangled polymer dynamics based on the Rouse or reptation model. Determination from unentangled motion is beneficial in terms of the computational efficiency. Generally, relaxation of unentangled motion is much faster than that of entangled motion. Thus, the scaling factor can be determined with less effort. Hermandaris et al. [18] compared the unentangled polymer dynamics of the atomistic model with that of the CG model by using the mean-squared displacements (MSDs) of the short chain motions, and they determined the temporal scaling factor based on the Rouse model. They observed hundreds of nanoseconds of polymer dynamics by quantified CGMD simulations of PS melts. Nevertheless, determination of the scaling factor based on the Rouse model should be carefully performed. The scaling law of the Rouse model is supported by the Gaussian nature of the motion of the segment with sufficiently high molecular weight (i.e., the long chain). However, several atomistic and CG models do not show Gaussian nature at $M<M_{c}$ [67,68]. The non-Gaussian nature becomes the cause of deviation of the scaling law from that of the Rouse model. Moreover, there is a concern that a lot of accurate CG models have non-Gaussian nature in the unentangled state. Saleno et al. [69] compared the polymer dynamics of the atomistic PE model with that of the coarse-grained PE model for different degrees of coarse graining. They found that a small degree of coarse graining (4–6 monomers per coarse-grained particle) is required to correctly describe the macroscopic behavior of the polymer properties. The small degree of coarse graining indicates non-Gaussian nature of the coarse-grained particle. Therefore, the deviation of the scaling law for unentangled motion should be carefully examined for each molecular model.

Another strategy is to use the scaling law of entangled motion. This is computationally expensive, but it is a promising method because some scaling laws are robust with respect to the degree of coarse graining for $M>M_{c}$. In other words, the entangled motion based on the reptation model surpasses the non-Gaussian nature of the segment that consists of the short chain. In this work, we determined the spatial and temporal scaling factors focusing on the entangled motion. Takahashi et al. [20] focused on the critical point of the scaling laws of the self-diffusion coefficient and end-to-end relaxation time with respect to the molecular weight. They determined a single set of scaling factors that attain the best accuracy for rescaling the KG model to the units and dimensions of the UA model of PE. However, their scheme was optimized for comparison of the above two models. To determine the scaling factors in a more general and robust way, we performed the following six steps: (i) for each molecular model, compute the scaling laws with respect to the molecular weight for the self-diffusion coefficient *D*–*M*, end-to-end relaxation time $\tau_{R}$–*M*, mean-squared end-to-end vector $\langle R^{2}\rangle$–*M*, and mean-squared radius of gyration $\langle R_{G}^{2}\rangle$–*M* (some other kinds of scaling law are basically addable), (ii) determine the critical molecular weight of each molecular model from the critical point of *D*–*M* or $\tau_{R}$–*M* scaling laws, and determine a scaling factor of the mass, $m^{*}=M_{c,\mathrm{AMD}}/M_{c,\mathrm{CGMD}}$ (see Table 1 for actual values), (iii) define the undetermined scaling factors for length and time, (iv) rescale the scaling laws of the CG models using the undetermined scaling factors, (v) calculate the relative root-mean-squared deviations (rRMSDs) between the scaling laws of the atomistic model and those of the rescaled CG model, and (vi) determine the set of spatial and temporal scaling factors that give the smallest sum of the rRMSDs. The definition of rRMSD is as follows:

(12) $$\begin{array}{c} {\mathrm{rRMSD}}_{X}^{2}\left(p,q\right)=\frac{1}{n-1}\sum_{M\geq M_{c}}{\left|\delta_{X}\left(p,q,M\right)\right|}^{2}, \end{array}$$

(13) $$\begin{array}{c} \delta_{X}\left(p,q,M\right)=\frac{X_{\mathrm{CGMD}}\left(p,q,m^{*}M\right)-X_{\mathrm{AMD}}\left(M\right)}{X_{\mathrm{AMD}}\left(M\right)} \end{array}$$

where ${\mathrm{rRMSD}}_{X}$ is the rRMSD of the scaling law for property *X*, *p* and *q* are undetermined scaling factors, *n* is the number of data points at $M\geq M_{c}$, and $\delta_{X}$ is the deviation of property *X* from the results of AMD.

## 3. Results and Discussion

### 3.1. Scaling Laws

**Figure 1:**
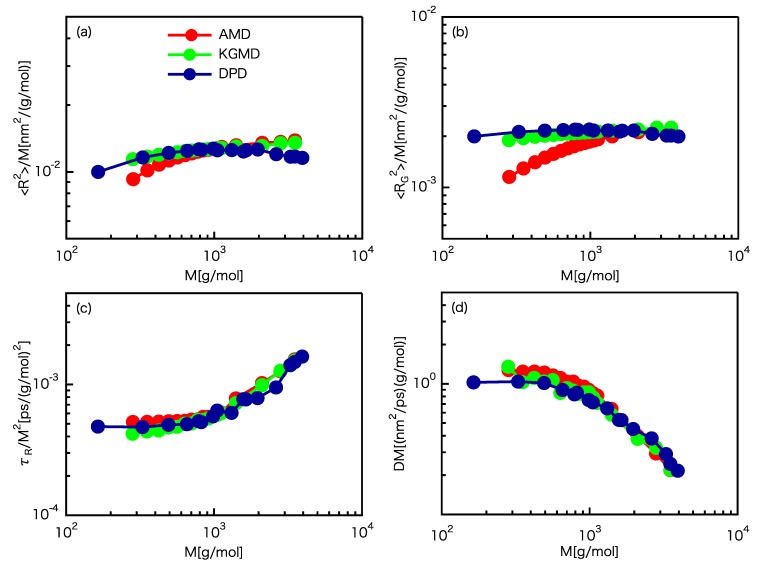
Comparison of the scaling laws for the UA PE model and rescaled CG models. (**a**) $\langle R^{2}\rangle$–*M* scaling law. (**b**) $\langle R_{G}^{2}\rangle$–*M* scaling law. (**c**) $\tau_{R}$–*M* scaling law. (**d**) *D*–*M* scaling law.

First of all, the *D*–*M*, $\tau_{R}$–*M*, $\langle R^{2}\rangle$–*M*, and $\langle R_{G}^{2}\rangle$–*M* scaling laws are computed. Takahashi et al. [20] shows that the critical molecular weight can be determined from the change in *D*–*M* and $\tau_{R}$–*M* power law exponents that indicate the onset of entanglement, and the determined value corresponds to the critical molecular weight estimated from primitive path analysis [70,71,72]. Therefore, we employ their strategy as a simple and robust way to determine the critical molecular weight. The $\langle R^{2}\rangle$–*M* and $\langle R_{G}^{2}\rangle$–*M* scaling laws are shown in Figure 1a,b, respectively. For $\langle R^{2}\rangle$–*M*, the rescaled KGMD results are almost equal to the AMD results for $M\geq M_{c}$, whereas the rescaled DPD results slightly deviate from the AMD results for $M\geq M_{c}$. However, for $\langle R_{G}^{2}\rangle$–*M*, the rescaled KGMD and DPD results are almost equal to the AMD results for $M\geq M_{c}$. The above results indicate that the determined spatial scaling factor has adequate accuracy. The $\tau_{R}$–*M* scaling law is shown in Figure 1c. The rescaled KGMD and DPD results are almost equal to the AMD results for $M\geq M_{c}$. This indicates that the temporal scaling factor is reasonable. The *D*–*M* scaling law is shown in Figure 1d. The rescaled KGMD and DPD results are almost equal to the AMD results for $M\geq M_{c}$. Please note that both the spatial and temporal scaling factors were required for rescaling *D*. The results indicate that the set of scaling factors is reasonable. The scaling factors determined from our scheme are given in Table 1.

### 3.2. Static Properties

In the following sections, scaling factors determined in the previous section (see Table 1) were used for rescaling the KGMD and DPD results to real units.

**Figure 2:**
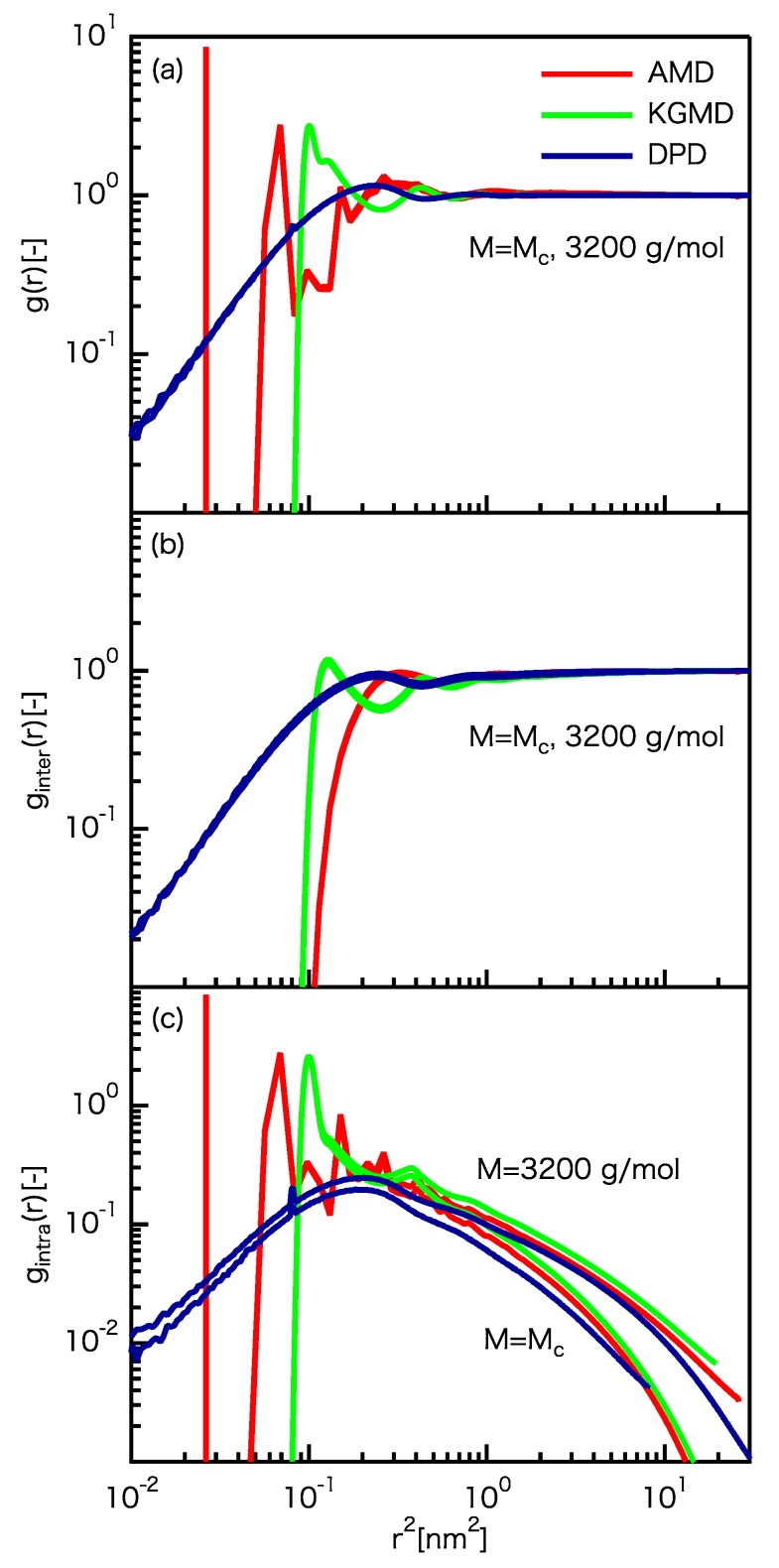
Comparison of the radial distribution function (RDFs) for the UA PE model and rescaled CG models. (**a**) RDF for all particles. (**b**) RDF for intermolecular particles. (**c**) RDF for intramolecular particles.

The scale gap can be approximately evaluated from the scaling factors, but its details should be carefully determined by comparison of the physical properties. To reveal the details of the spatial scale gap, some static properties of the MD simulations were compared. The radial distribution function g(r) is useful to reveal the local structure of polymer melts:

(14) $$\begin{array}{c} g\left(r\right)=\frac{1}{4\pi r^{2}\Delta r\rho }\frac{\langle \sum_{j}n_{j}\left(r\right)\rangle }{N_{\mathrm{total}}-1}, \end{array}$$

where ρ is the number density of particles and $n_{j}\left(r\right)$ is the number of particles (i.e., carbon atoms/beads) in the region between *r* and r+Δr when particle *j* is on r=0. The term $n_{j}\left(r\right)$ can be defined for the total, intermolecular, and intramolecular contributions. g(r) describes the distance dependence of the probability for beads/carbon atoms to meet. g(r) for all particles, intermolecular particles, and intramolecular particles is shown in Figure 2a–c, respectively. For all of the results, the peaks of the three models do not coincide at r<0.77 nm, but the plateaus/curves at r>0.77 nm do coincide. For DPD, violation of the excluded volume effect is observed, showing that the DPD particle can pass other DPD particles even after implementing slip-springs.

**Figure 3:**
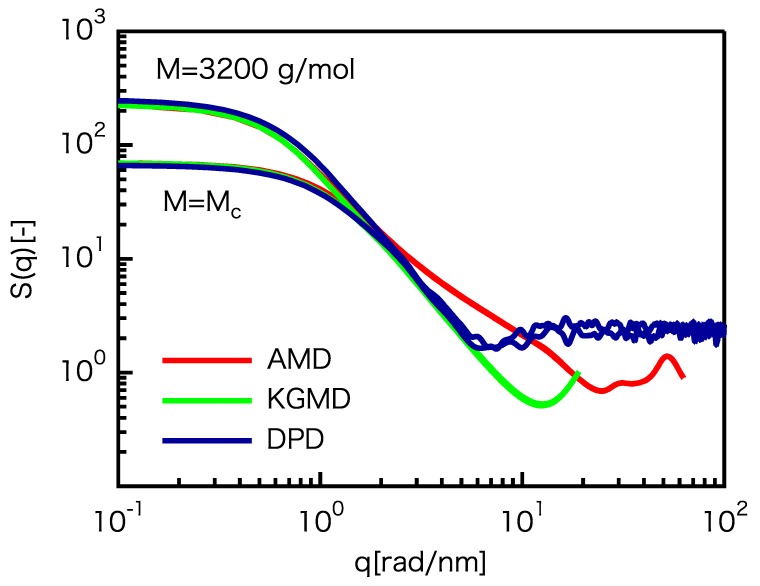
Comparison of the static structure factors for the UA PE model and rescaled CG models.

The static structure factor of an individual chain S(q) is useful to determine the small and large internal structures of polymer melts:

(15) $$\begin{array}{c} S\left(q\right)=1+\frac{1}{N_{\mathrm{total}}}\langle \sum_{j\neq k}\exp\left[-iq\cdot \left(r_{j}-r_{k}\right)\right]\rangle , \end{array}$$

where *q* is the spatial frequency equal to 2π/r. The fractal scattering of $S\left(q\right)∼q^{-1/\nu }$ is expected to be equal to $q^{-2}$ (ν=1/2) and independent of the chain length. The static structure factors for the three models are shown in Figure 3. The S(q) curves coincide for q<2.0 rad/nm, but the fractal scattering of the CG models is clearly different from that of the atomistic model for q>2.0 rad/nm. For the CG models, the fractal scattering for 1.0 rad/nm <q< 5.0 rad/nm is almost the same as that for an ideal chain. In contrast, the fractal scattering of the atomistic model for 2.0 rad/nm <q< 10 rad/nm is clearly different from the ideal value. This indicates that the expected cancellation of the dispersion forces for polymer melts [73] is not entirely satisfied for the atomistic model. The fractal scattering of the atomistic model for 2.0 rad/nm <q< 10 rad/nm is estimated to be $q^{-1.3}$. Please note that the fractal scattering of the atomistic model becomes almost ideal for 1.0 rad/nm <q<2.0 rad/nm, indicating the onset of cancellation of the dispersion forces at q=2.0 rad/nm (i.e., r=3.1 nm). In other words, S(q) of the atomistic model and CG models do not coincide at length scales smaller than 3.1 nm owing to the effect of dispersion forces. Even though the LJ interactions were cut off beyond 1.2 nm, internal structures up to 3.1 nm were subject to the influence of dispersion forces.

For the static properties, the largest threshold value of length is 3.1 nm. This is approximately equal to ${\langle R^{2}\rangle }^{1/2}$ at $M=M_{c}$, indicating mismatch of the static structures between the CG models and the atomistic model within the distance between the strands of the polymer network.

### 3.3. Dynamic Properties

**Figure 4:**
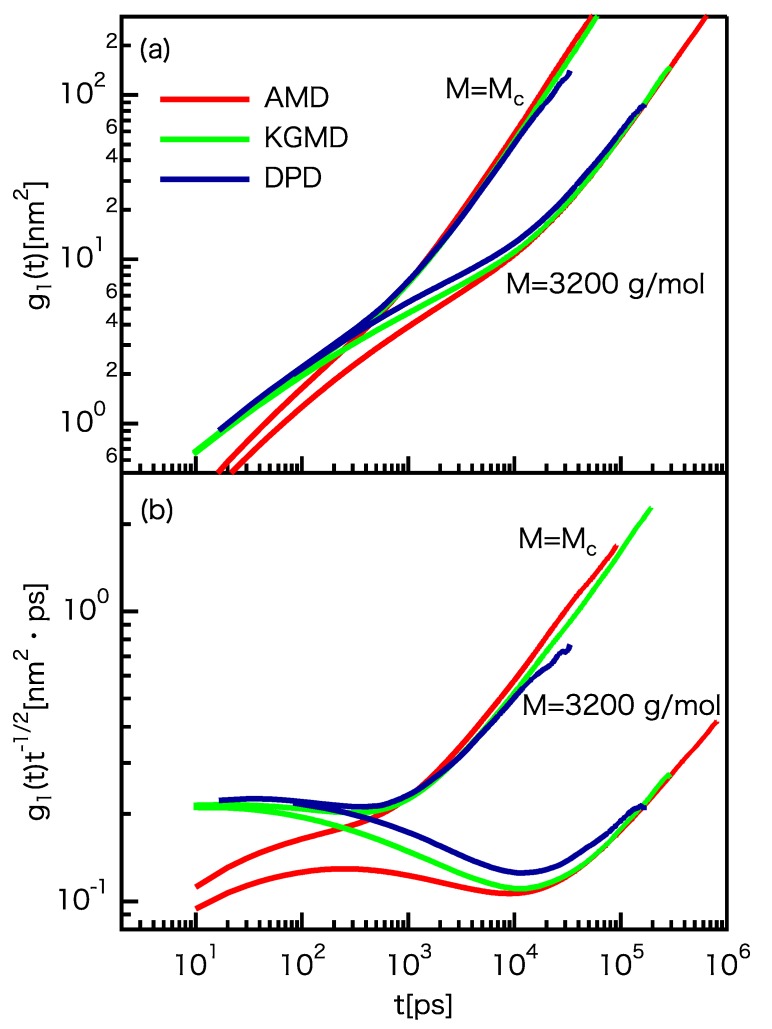
Comparison of the MSD of the chain center $g_{1}\left(t\right)$ for the UA PE model and rescaled CG models. (**a**) $g_{1}\left(t\right)$. (**b**) $g_{1}\left(t\right)t^{-1/2}$.

To clarify the details of the temporal scale gap, some dynamic properties of the MD simulations were compared. The MSD of the chain center $g_{1}\left(t\right)$ shows the relaxation motion of mass transfer:

(16) $$\begin{array}{c} g_{1}\left(t\right)=\langle {\left[r_{c.c.}\left(t\right)-r_{c.c.}\left(0\right)\right]}^{2}\rangle , \end{array}$$

where $r_{c.c.}$ is the coordinates of the chain center. From the Rouse and reptation models, the scaling-law sequence for the MSD is expected to be approximately

(17) $$\begin{array}{c} g_{1}\left(t\right)∼\left\{\begin{array}{c} t^{1}\;\left(t<\tau_{0}\right)\\ t^{1/2}\;\left(\tau_{0}<t<\tau_{c}∼N_{c}^{2}\right)\\ t^{1/4}\;\left(\tau_{c}<t<\tau_{N}∼N^{2}\right)\\ t^{1/2}\;\left(\tau_{N}<t<\tau_{R}∼N^{3}/N_{c}\right)\\ t^{1}\;\left(t>\tau_{R}\right) \end{array}\right., \end{array}$$

where $N_{c}$ is the segment number corresponding to $M_{c}$, *N* is the segment number per chain, $\tau_{0}$ is a specific short time, and $\tau_{c}$ and $\tau_{N}$ are the Rouse relaxation times that correspond to $N_{c}$ and *N*, respectively. The $g_{1}\left(t\right)$ curves for the three models are shown in Figure 4a. For $M=M_{c}$(=980 g/mol), the $g_{1}\left(t\right)$ curves coincide for t>550 ps. For M=3200 g/mol, the $g_{1}\left(t\right)$ curves coincide for t> 10,000 ps. These threshold values of 550 ps and 10,000 ps are proportional to $\tau_{R}∼N^{3}∼M^{3}$, showing the delay in the onset of dynamic universality for the polymer melts. The matching between the KG and atomistic models is slightly better than that between the DPD and atomistic models. However, the shape of the $g_{1}\left(t\right)$ curve for the DPD model is close to that of the KG model. The threshold values of Equation (17) are unclear from the $g_{1}\left(t\right)$ results themselves. Thus, we plotted $g_{1}\left(t\right)t^{-1/2}$ against time (Figure 4b). The scaling law for $\tau_{N}<t<\tau_{R}∼N^{3}/N_{c}$ is approximately satisfied at about t= 10,000 ps, irrespective of the differences of the models. In contrast, the scaling laws of the CG models for $t<\tau_{N}$ deviate from those of the atomistic model. This indicates the difficulty in determining accurate scaling factors from the MSDs of short chain motions. The shape of the $g_{1}\left(t\right)t^{-1/2}$ curve for the DPD model is close to that of the KG model, indicating the similarity of the short chain motions of the two models. Please note that *D* can be determined from $g_{1}\left(t\right)$ because $g_{1}\left(t\right)∼t^{1}$ is expected for $t>\tau_{R}$.

The time-correlation function of the end-to-end vector C(t) shows the relaxation motion of the longest chains:

(18) $$\begin{array}{c} C\left(t\right)=\frac{\langle R(t)\cdot R(0)\rangle }{\langle R^{2}\rangle }. \end{array}$$

**Figure 5:**
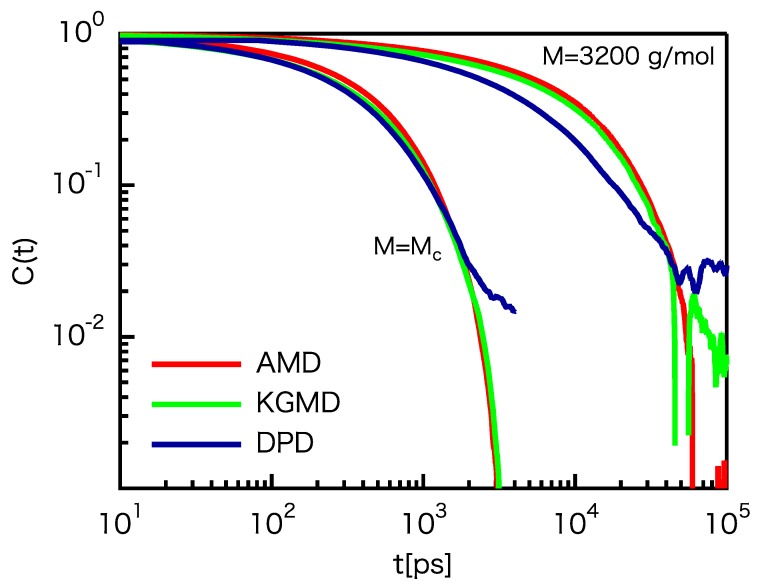
Comparison of the time-correlation functions of the end-to-end vector for the UA PE model and rescaled CG models.

Logarithmic plots of C(t) for the three models are shown in Figure 5a. For $M=M_{c}$, the C(t) curves coincide for t<100 ps. For M=3200 g/mol, the C(t) curves coincide for t<1000 ps. However, the DPD results at M=3200 g/mol are slightly smaller than the others for t>1000 ps. This behavior corresponds to the results of $g_{1}\left(t\right)$. For DPD, the $g_{1}\left(t\right)$ value is slightly larger than the others, i.e., mass transfer is slightly faster.

Please note that $\tau_{R}$ can be determined from C(t) because it is expected that $C\left(t\right)∼\exp\left(-t/\tau_{R}\right)$.

**Figure 6:**
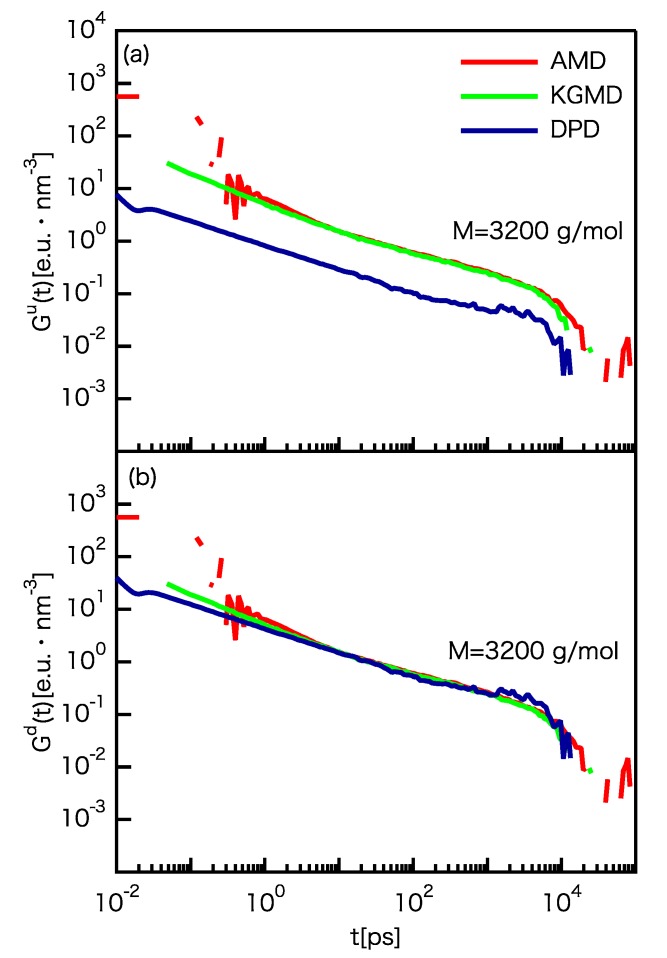
Comparison of G(t) for the UA PE model and rescaled CG models. (**a**) G(t) rescaled by the scaling factor of energy per volume (${\left(k_{B}T\right)}^{*}/l^{*3}$) based on the unit of the relaxation modulus. (**b**) G(t) using the scaling factor determined by Equation (22).

The relaxation modulus G(t) shows the viscoelastic behavior of polymer melts:

(19) $$\begin{array}{c} G\left(t\right)=\frac{V}{k_{B}T}\langle \sigma_{\alpha \beta }\left(t\right)\sigma_{\alpha \beta }\left(t\right)\rangle , \end{array}$$

where $\sigma_{\alpha \beta }$ are the off-diagonal stress components xy, xz, and yz. For the rheological property, it is generally believed that the scaling factor should be determined from the rheological response. Indeed, mismatch of the scaling factor was demonstrated between direct determination from comparison of the rheological response and the use of the unit of G(t) (energy per volume). For convenience, we defined two different types of *G* scaling factors: the *G* scaling factor from direct comparison $G^{d}$ and the *G* scaling factor after using the unit $G^{u}$. Thus, $G^{d}$ and $G^{u}$ satisfy the following relation:

(20) $$\begin{array}{c} G^{u}=G^{d}\frac{{\left(k_{B}T\right)}^{*}}{l^{*3}}, \end{array}$$

where ${\left(k_{B}T\right)}^{*}$ is the scaling factor for $k_{B}T$. The $G^{d}$ and $G^{u}$ values are given in Table 1. G(t) rescaled by the scaling factor of energy per volume (${\left(k_{B}T\right)}^{*}/l^{*3}$) based on the unit of the relaxation modulus is shown in Figure 6a. The KGMD results are almost the same as the AMD results, despite not using $G^{d}$. This is because of the similarity of the polymer networks. For rubber elasticity theory, the shear modulus of polymer networks can be determined by

(21) $$\begin{array}{c} G=A\nu k_{B}T=A\rho {\left(N_{e}^{\mathrm{PPKuhn}}\right)}^{-1}k_{B}T, \end{array}$$

where ν and ρ are the number density of the network strand and segment, respectively, *A* is a constant dependent on the network functionality and fluctuations imposed to the network nodes, and $N_{e}^{\mathrm{PPKuhn}}$ is the segment number between entanglements. Please note that $N_{e}^{\mathrm{PPKuhn}}$ is equal to $N_{c}$, which is referred to as the “Kuhn length of the primitive path” and can be determined not only by the scaling law but also by primitive path analysis [70,71,72]. From Equations (20) and (21) and Table 1, $G^{d}$ can be rewritten as

(22) $$\begin{array}{c} G^{d}=A^{*}\nu^{*}\frac{l^{*3}}{{\left(k_{B}T\right)}^{*}}=A^{*}\rho^{*}N_{c}^{*-1}\frac{l^{*3}}{{\left(k_{B}T\right)}^{*}}. \end{array}$$

Thus, the similarity of the polymer networks can be examined using the *A* and ν values. For KGMD, the ν value is 0.85 times that of AMD, indicating that the strand densities of the two model are approximately the same (see Table 1). From Equation (22) and $G_{u}=1.0$, the $A^{*}$ value was determined to be 1.2, showing that the polymer network of the KG model is very similar to that of the atomistic model. In contrast, the G(t) results of DPD greatly deviate from those of AMD. For DPD, the ν value is 1.3 times that of AMD, indicating that the strand densities of the two models are relatively close. However, $G_{u}$ between AMD and DPD is 0.19. Thus, the $A^{*}$ value is 0.14, showing that the polymer network of the DPD model is considerably different from that of the atomistic model. Nevertheless, using Equation (22), the G(t) results successfully rescaled for DPD. G(t) using the scaling factor determined by Equation (22) is shown in Figure 6b. All of the results coincide for t>10 ps.

For the dynamic properties, the largest threshold value of time is proportional to $M^{3}$, showing the delay in the onset of dynamic universality for the polymer melts. This differs from the onset of static universality in terms of whether the observed threshold value continues to depend on the chain length. If the threshold value of time continues to depend on the chain length, it becomes difficult to effectively switch the use of AMD and CGMD with respect to the timescale. The delay in the onset of dynamic universality may disappear for relatively large molecular weight, but this condition is beyond the capability of current computers.

### 3.4. Computational Efficiency

The computational cost of MD simulations can generally be estimated by the number of particles in the simulation system $N_{p}$ and the number of times of numerical integration $N_{i}$. Recent advances in calculation methods have made it possible to reduce the computational cost of interaction calculations by up to $O(N_{p})$ while maintaining the accuracy, where *O* is a function indicating the order. Thus, the total computational cost of a MD simulation can be determined by $O(N_{p}N_{i})$. When considering numerical integration up to the same timescale for systems with the same particle scale, the relations of the total time step and number of particles between CGMD and AMD can be expressed as

(23) $$\begin{array}{ccc} {\left(N_{i}\Delta t\right)}_{\mathrm{CGMD}} & = & {\left(N_{i}\Delta t\right)}_{\mathrm{AMD}}\cdot t^{*}, \end{array}$$

(24) $$\begin{array}{ccc} N_{p,\;\mathrm{CGMD}} & = & N_{p,\;\mathrm{AMD}}\cdot N^{*}. \end{array}$$

Thus, the ratio of the total computational cost of CGMD to that of AMD can be estimated by

(25) $$\begin{array}{ccc} \frac{O{\left(N_{i}N_{p}\right)}_{\mathrm{CGMD}}}{O{\left(N_{i}N_{p}\right)}_{\mathrm{AMD}}} & = & \frac{\Delta t_{\mathrm{AMD}}}{\Delta t_{\mathrm{CGMD}}}\frac{1}{N^{*}t^{*}}, \end{array}$$

where Δt is the time step for numerical integration of the equation of motion. For KGMD, the total computational cost is 4.2 times that of AMD, indicating that the computational efficiency of the KG model is lower than that of the UA model. This is partly because of that the number of beads (or site) in single chain of KG model is same as that of UA model. Ordinary, CG models for polymer chains supposes that one segment of CG models contains several repeat units. In this work, however, “repeat units per segment” is simply determined from mass (or carbon number) scaling factor shown in Table 1. Therefore “one unit per segment” is basically possible, and an inversion of computational efficiency is also possible eventuality. The inversion of computational efficiency between UA and KG model in this work can be thought of as an overestimating of arbitrary parameter Γ in KGMD. The large Γ makes dynamics slow because the friction between chains becomes large [74]. It can be assumed that the Γ of AMD using TraPPE-UA is considerably smaller than that of KGMD. This fact indicates an existence of real case that the optimization of basic parameter of KG model becomes necessary for efficient coarse graining. For DPD, the total computational cost is 0.52 times that of AMD. Thus, coarse graining of the UA model using the DPD model has the benefit of speeding up the MD simulations by about 2 times. The DPD with slip-spring model can be considered to be an option for coarse graining of the UA PE model.

In Table 2 we summarize the overall performance of UA, KG, and DPD models to simulate PE melts.

## 4. Conclusions

We investigated the scale gaps among the atomistic model of PE melts, bead–spring KG model, and DPD with slip-spring model. First, the single set of spatial and temporal scaling factors between the atomistic model and each CG model was determined. The method to determine the scaling factors was systematic and robust because some representative scaling laws of straight-chain polymer melts that can be determined from experimental/computational results were used. The rescaled scaling laws of the CG models coincide with that of the atomistic model for $M>M_{c}$, showing that the entangled motion is more robust than the non-Gaussian nature of the segment which consists of the short chain. The results of the CGMD simulations were rescaled using the well-estimated scaling factors and compared with those of the atomistic model. Threshold values that indicate the onset of the static and dynamic universality among the polymer models were obtained for almost all of the results of comparison of the polymer properties. For the static properties, the largest threshold value of the length is 3.1 nm. This value is approximately equal to ${\langle R^{2}\rangle }^{1/2}$ at $M=M_{c}$, indicating the mismatch of the static structures between the CG models and the atomistic model within the distance between the strands of the polymer network. For the dynamic properties, the largest threshold value of time is proportional to $M^{3}$, showing the delay in the onset of the dynamic universality for the polymer melts. This is in contrast to the onset of static universality in terms of whether the observed threshold value continues to depend on the chain length. If the threshold value of time continues to depend on the chain length, it becomes difficult to effectively switch the use of AMD and CG MD with respect to the timescale. The delay in the onset of dynamic universality may disappear for relatively large molecular weight, but this condition is beyond the capability of current computers. From the G(t) results, and thus the *A* value, the similarity of the polymer networks among the molecular models was quantitatively evaluated. The KG model has a similar polymer network to the atomistic model, whereas the polymer network of the DPD model considerably different from that of the atomistic model. The computational efficiency of each CG model was quantitatively estimated with respect to the atomistic model. The total computational cost of KGMD is 4.2 times that of AMD, whereas that of DPD is 0.52 times that of AMD. The KG model is clearly a poor choice for coarse graining of the UA PE model, whereas the DPD with slip-spring model is a possible choice for coarse graining, and it has the benefit of speeding up the MD simulations by about two times.

The performance of the CG models of polymers was systematically and quantitatively evaluated in terms of both the accuracy and computational efficiency. Using our scheme, the following three points can be quantitatively evaluated for CG models: (i) the “scale gap” of the static and dynamic properties from that of the atomistic model, (ii) the similarity of the polymer network to that of the atomistic model, and (iii) the computational efficiency relative to the atomistic model. The above three points are crucial elements for further development of better CG models and/or coarse-graining schemes. In future studies, the performance of other CG models will be extensively evaluated using our scheme.

## Figures and Tables

**Table 1:** Scaling factors of the UA model of PE melts, KG model, and DPD with slip-spring model. ${}^{a}$ “Carbon num.” becomes the “dimension” of segment number. ${}^{b}$ 1 e.u. = 6.9 × 10${}^{-21}$ J. ${}^{c}$ 1 e.u./nm${}^{3}$ = 6.9 × 10${}^{6}$ J/m${}^{3}$ = 6.9 MPa.

Property	Dimension	Symbol for	UA	(Unit)	KG	(Unit)	DPD	(Unit)
		Scaling Factor						
—	Length	$l^{*}$	1	nm/nm	0.33	nm/σ	0.57	nm/$r_{c}$
—	Time	$t^{*}$	1	ps/ps	0.080	ps/$\tau_{\mathrm{KG}}$	0.028	ps/$\tau_{\mathrm{DPD}}$
—	Mass	$m^{*}$	1	g/mol/(g/mol)	14	g/mol/$m_{\mathrm{KG}}$	32	g/mol/$m_{\mathrm{DPD}}$
—	Energy	—	1	J/J	6.9$\times 10^{-21}$	J/${\left(k_{B}T\right)}_{\mathrm{KG}}$	6.9$\times 10^{-21}$	J/${\left(k_{B}T\right)}_{\mathrm{DPD}}$
—	(Carbon num.) ${}^{a}$	$N^{*}$	1	(carbons/carbons)	1.0	(carbons/KG segs.)	2.3	(carbons/DPD segs.)
$k_{B}T$	Energy	${\left(k_{B}T\right)}^{*}$	1	e.u./e.u. ${}^{b}$	1.0	e.u./e.u.	1.0	e.u./e.u.
Segment density	(Carbon num.)/Length${}^{3}$	$\rho^{*}$	1	$\frac{{(\mathrm{carbons})\mathrm{nm}}^{-3}}{{(\mathrm{carbons})\mathrm{nm}}^{-3}}$	0.85	$\frac{{(\mathrm{carbons})\mathrm{nm}}^{-3}}{{(\mathrm{carbons})\mathrm{nm}}^{-3}}$	1.3	$\frac{{(\mathrm{carbons})\mathrm{nm}}^{-3}}{{(\mathrm{carbons})\mathrm{nm}}^{-3}}$
Critical segment number	(Carbon num.)	$N_{c}^{*}$	1	(carbons/carbons)	1.0	(carbons/carbons)	1.0	(carbons/carbons)
Strand density	Length${}^{-3}$	$\nu^{*}=\rho^{*}/N_{c}^{*}$	1	nm${}^{-3}$/nm${}^{-3}$	0.85	nm${}^{-3}$/nm${}^{-3}$	1.3	nm${}^{-3}$/nm${}^{-3}$
$G^{d}$	Energy/Length${}^{3}$	(See Equation (22))	1	$\frac{e.u.\cdot {\mathrm{nm}}^{-3}}{e.u.\cdot {\mathrm{nm}}^{-3}}$ ${}^{c}$	0.036	$\frac{e.u.\cdot {\mathrm{nm}}^{-3}}{e.u.\cdot {\mathrm{nm}}^{-3}}$	0.036	$\frac{e.u.\cdot {\mathrm{nm}}^{-3}}{e.u.\cdot {\mathrm{nm}}^{-3}}$
$G^{u}$	Energy/Length${}^{3}$	$G^{u}$	1	$\frac{e.u.\cdot {\mathrm{nm}}^{-3}}{e.u.\cdot {\mathrm{nm}}^{-3}}$	1.0	$\frac{e.u.\cdot {\mathrm{nm}}^{-3}}{e.u.\cdot {\mathrm{nm}}^{-3}}$	0.19	$\frac{e.u.\cdot {\mathrm{nm}}^{-3}}{e.u.\cdot {\mathrm{nm}}^{-3}}$
*A*	—	$A^{*}$	1	—/—	1.2	—/—	0.14	—/—

**Table 2:** Comparison of overall performance of molecular models for PE melts simulations. “A”, “B”, “C”, and “D” mean “very good”, “good”, “adequate”, and “no good” performance, respectively. ${}^{a}$ “very good” after using Equation (22).

		UA	KG	DPD
Static properties	$\langle R^{2}\rangle$	A	B	C
	$\langle R_{G}^{2}\rangle$	A	C	C
	g(r)	A	B	B
	S(q)	A	B	B
Dynamic properties	*D*	A	A	A
	$\tau_{R}$	A	A	B
	$g_{1}\left(t\right)$	A	B	B
	C(t)	A	B	C
	G(t)	A	A	A ${}^{a}$
Computational cost		C	D	B
